# Intravitreal injection of the Galectin-3 inhibitor TD139 provides neuroprotection in a rat model of ocular hypertensive glaucoma

**DOI:** 10.1186/s13041-024-01160-z

**Published:** 2024-11-22

**Authors:** Anne Rombaut, Rune Brautaset, Pete A. Williams, James R. Tribble

**Affiliations:** Department of Clinical Neuroscience, Division of Eye and Vision, St. Erik Eye Hospital, Karolinska Institutet, Stockholm, Sweden

**Keywords:** Galectin-3, TD139, Neurodegeneration, Neuroinflammation, Neuroprotection, Retina, Retinal ganglion cells, Astrocytes, Microglia, Glaucoma

## Abstract

Neuroinflammation is a significant contributor to the pathology of glaucoma. Targeting key-mediators in this process is a realistic option to slow disease progression. Galectin-3 is a β-galactoside binding lectin that has been associated with inflammation in both systemic and central nervous system diseases. Elevated Galectin-3 has recently been detected in multiple animal models of glaucoma and inhibiting Galectin-3 using an intravitreal injection of TD139 (a Galectin-3 small molecule inhibitor) is neuroprotective. We queried whether this neuroprotective effect was translatable to another animal model and species. TD139 was intravitreally injected, in a rat ocular hypertensive model of glaucoma, 3 days after the induction of ocular hypertension (at peak intraocular pressure). Retinal ganglion cell survival and glial morphological markers were quantified. The degeneration of retinal ganglion cells was prevented by TD139 injection, but gross glial markers remained unaffected. These data confirm that the intravitreal injection of TD139 is neuroprotective in a rat ocular hypertensive model of glaucoma, while suggesting that the inhibition of Galectin-3 is not sufficient to alter the gross inflammatory outcome.

**Supplementary Information:**

The online version contains supplementary material available at 10.1186/s13041-024-01160-z.

Glaucoma is a progressive, neurodegenerative disease resulting in the degeneration of retinal ganglion cells (RGCs), the output neurons of the retina via the optic nerve. There is increasing evidence in both animal models of glaucoma and human glaucoma patients that neuroinflammation plays a pivotal role in the disease pathology [1, 2]. One protein that has been consistently linked to inflammation in both systemic and central nervous system diseases is the β-galactoside binding lectin; Galectin-3 (Gal-3, also known as Mac-2) [3, 4]. Gal-3 has been shown to activate microglia [5], act as a chemoattractant for monocytes [6], and is a marker of pro-phagocytotic states. Astrocytes in the optic nerve demonstrate strong expression of Gal-3 and this is increased during glaucomatous neurodegeneration [7, 8]. Margeta et al. demonstrated that Gal-3 was upregulated in activated microglia in two different mouse models of glaucoma (inducible ocular hypertension and the DBA/2J mouse), and that genetic knockout (*Lgals3*^*−/−*^) or pharmacological inhibition of Gal-3 via intravitreal injection of the small molecule inhibitor TD139 had a neuroprotective effect on RGCs [9]. We previously identified an increase in Gal-3 in a rat bead model of glaucoma at a degenerative timepoint [10]. We sought to determine whether intravitreal injection of TD139 could also provide neuroprotection in the rat bead model of glaucoma, which would support the potential for TD139 translation if successful across species and models.

Ocular hypertension (OHT) was induced bilaterally in Brown Norway rats via an injection of magnetic beads as described previously [11]. OHT rats or normotensive controls received a single 3 µL intravitreal injection of TD-139 at 50 ng/µL in 5% DMSO in HBSS (Selleck Chemicals LLC, cat. S0471) or 5% DMSO in HBSS (vehicle control), 3 days after OHT induction (when IOP is high and early pro-inflammatory responses are evident [10]). Intraocular pressure was recorded every 2–3 days by rebound tonometry in awake rats. Rats were euthanized 14 days after OHT induction and eyes were processed as flatmounts for immunofluorescent labeling of RNA-binding protein with multiple splicing (RBPMS; RGC specific marker), Isolectin GS-IB4 (IB4; monocyte, microglia, and vasculature marker), and glial fibrillary acidic protein (GFAP) (astrocyte / Müller cell marker) (Table S1), as described previously [10], with the exception that here 2% bovine serum albumin in PBS was used. Single plane or *Z*-stack confocal images were acquired and RBPMS + RGCs and IB4 + microglia or monocytes were counted using the Cell counter plug-in in FIJI (nerve fiber layer (NFL)-ganglion cell layer (GCL) and the inner plexiform layer (IPL)-inner nuclear layer (INL) were separated). Individual IB4 + microglia surface areas were measured with the FIJI Polygon selection tool. The volume of GFAP was reconstructed in Imaris using the Surface tool. Statistical analysis was performed in R. All tests except for the IB4 + surface area analysis were one-way *ANOVA* followed by *Dunnett’s* multiple comparison *post hoc* test. *Kruskal–Wallis* one way analysis of variance followed by pairwise *Wilcoxon rank sum* test was used to analyze non-normally distributed data. A mixed effect model was used for the analysis of IB4 + microglia surface areas as described previously [10].

**Fig. 1:**
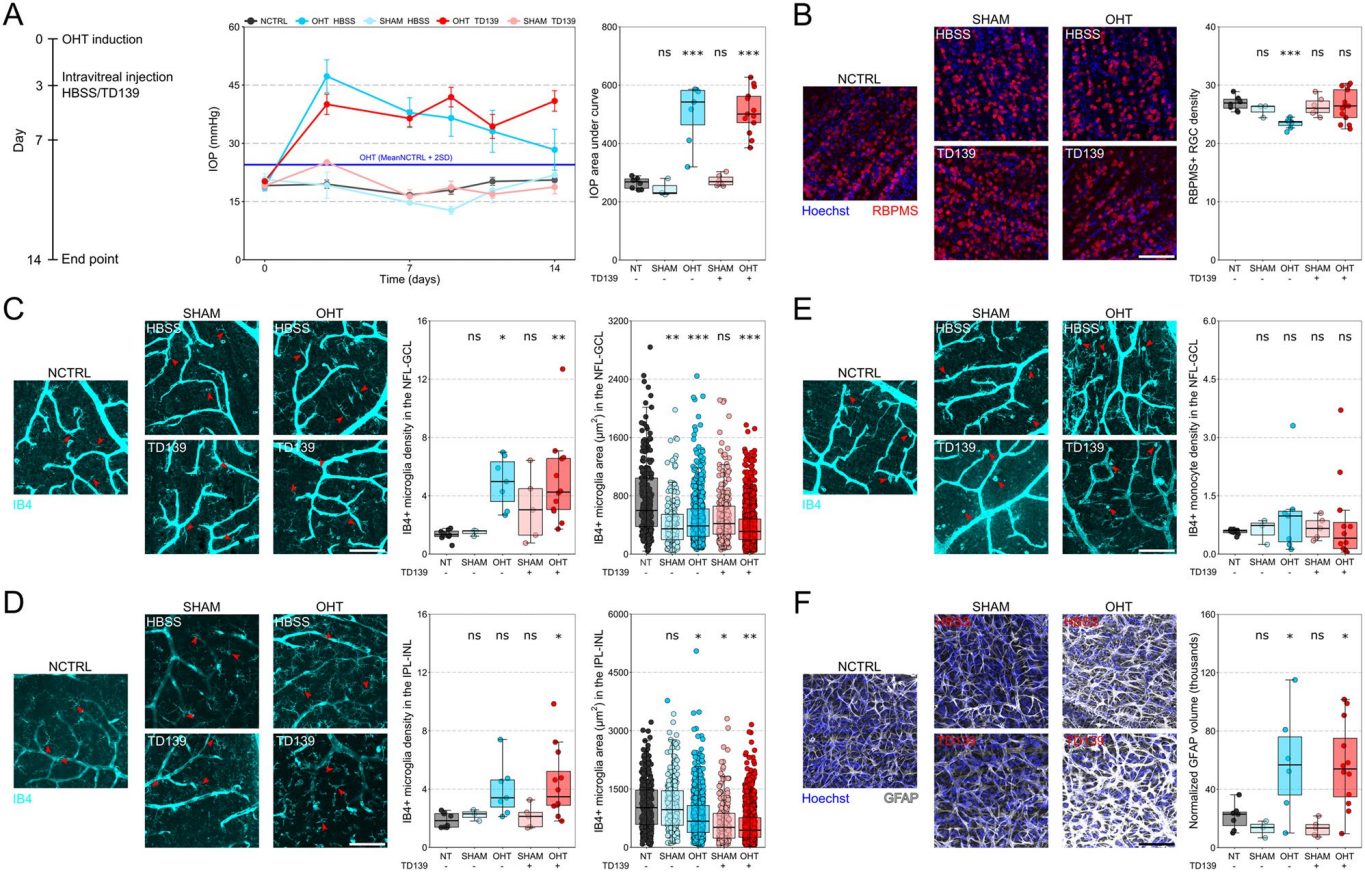
Intravitreal injection of TD139 results in neuroprotection but does not limit gross inflammation. **A**) Rats received TD139 (or vehicle) intravitreally 3 days after the induction of OHT. The IOP profile over time and area under the curve was comparable for OHT-HBSS and OHT-TD139. **B**) The density of RGCs (RBPMS+) was significantly lower for the OHT-HBSS group compared to the normotensive control (NCTRL) group, which was prevented by TD139 injection. **C**) In the NFL-GCL, the density of microglia (IB4+; *examples denoted by red arrows*) was significantly higher for the OHT-HBSS and OHT-TD139 group compared to the NCTRL group, and microglia surface area was significantly lower for all groups except for the SHAM-TD139 group compared to the NCTRL group. **D**) In the IPL-INL, microglia (IB4+; *examples denoted by red arrows*) density was significantly higher for the OHT-TD139 group compared to the NCTRL group, and microglia surface area was significantly lower for all groups except for the SHAM-HBSS group compared to the NCTRL group. **E**) The monocyte (IB4+; *examples denoted by red arrows*) density in the NFL-GCL did not change significantly for any of the groups compared to the NCTRL group. **F**) Astrocyte (GFAP+) volume in the NFL was significantly higher for the OHT-HBSS and OHT-TD139 group compared to the NCTRL group. ns = *P* > 0.05, * = *P* < 0.05, ** = *P* < 0.01, *** = *P* < 0.001. For B-F, scale bar = 100 μm. For B, C-D (left panels), and E-F data are normalized to 0.01 mm^2^ of retinal surface area. Conditions were: NCTRL (naïve normotensive controls; no bead injection, no intravitreal injection; *n* = 8 retina), SHAM-HBSS (sham controls; bead injection without OHT, intravitreal injection of vehicle; *n* = 3 retina), SHAM-TD139 (drug controls; bead injection without OHT, intravitreal injection of TD139; *n* = 6 retina), OHT-HBSS (OHT controls; bead injection resulting in OHT, intravitreal injection of vehicle; *n* = 7 retina), and OHT-TD139 (OHT drug treated; AC injection resulting in OHT, intravitreal injection of TD139; *n* = 13 retina)

OHT-HBSS and OHT-TD139 demonstrated robust and sustained IOP increase with significantly increased mean and IOP area under the curve compared to naïve controls (NCTRL; all *P* < 0.5, Fig. 1A; **Supp** Fig. 1; Table 2). OHT-HBSS resulted in significant loss of RGC density compared to NCTRL (*P* = 0.003; Fig. 1B; Table S2) which was robustly protected against by TD139 (*P* = 0.656 compared to NCTRL). SHAM-TD139 had no effect on IOP metrics (all *P* < 0.05, Fig. 1A; **Supp** Fig. 1; Table S2) or RGC survival compared to NCTRL (*P* = 0.609, Fig. 1B; Table S2). This confirmation of neuroprotection across additional species supports the potential of TD139 as a glaucoma therapeutic.

Given Gal-3’s known role in pro-inflammatory and disease-associated microglia we next assessed the effect of TD139 treatment on glial morphology, a useful surrogate of inflammatory state. In the NFL-GCL, IB4 + microglia density was significantly increased and surface area was significantly reduced in OHT-HBSS (*P* = 0.011 and *P* < 0.001 respectively, Fig. 1C; Table S2) consistent with microglial proliferation and retraction of processes. OHT-TD139 had no effect on microglial responses with similar density and morphology changes (*P* = 0.003 and *P* < 0.001 respectively, compared to NCTRL, Fig. 1C; Table S2). SHAM-TD139 did not result in significant change to microglial density or surface area, although there was some increased variability compared to NCTRL (*P* = 0.385 and *P* = 0.089 respectively, compared to NCTRL, Fig. 1C; Table S2). These effects were similar, yet less exaggerated, for microglia in the IPL/INL. Microglia density was not significantly increased, but was more variable, while surface area was significantly reduced in OHT-HBSS (*P* = 0.085 and *P* = 0.012 respectively, Fig. 1D; Table S2). Again, TD139 had no protective effect on this response, with microglial density significantly increased and surface area significantly reduced relative to NCTRL (*P* = 0.012 and *P* < 0.001 respectively, Fig. 1D; Table S2). SHAM-TD139 did not result in changes to microglia density in the IPL/INL but did result in significantly reduced surface area (*P* = 0.998 and *P* = 0.020 respectively, Fig. 1D; Table S2).

Infiltration of monocytes is a feature of this model and other models of experimental glaucoma [10, 12]. However, we did not detect a significant increase in monocytes in any of the conditions (*P* > 0.05 for all comparisons to NCTRL; Fig. 1E; Table S2).

Astrogliosis in the NFL is also a consistent feature of glaucoma models. Volume reconstruction of the GFAP signal in the NFL demonstrated that GFAP content was significantly increased for the OHT-HBSS and OHT-TD139 groups compared to the NCTRL group (*P* = 0.040, and *P* = 0.019, respectively, Fig. 1F; Table S2), whereas for the SHAM-HBSS and SHAM-TD139 groups no significant difference was detected (*P* = 0.970, and *P* = 0.957, respectively, Fig. 1F; Table S2). Collectively, these data indicate ongoing neuroinflammation in the OHT groups, that is not altered by intravitreal TD139.

These data demonstrate that a single, interventional, intravitreal injection of TD139 provides IOP-independent neuroprotection in the rat bead model of glaucoma. There is no amelioration of gross glial changes, suggesting that TD139’s effects are unlikely mediated through gross suppression of glial inflammatory responses, but does not preclude the attenuation of specific pathways. TD139 is a small molecule inhibitor which binds to the carbohydrate recognition domain of Gal-3 [13]. This domain enables Gal-3 interaction with a wide array of targets through which it can exert effects. Further studies which dissect these potential Gal-3 pathways are needed to determine the molecular effects of TD139 on retinal glia. These data do not undermine a pro-inflammatory effect of Gal-3 but suggest that targeting only Gal-3 is not sufficient to halt neuroinflammation in glaucoma. This may be better achieved as a combinational treatment with other anti-inflammatory or therapeutic agents. Nonetheless, the lack of a clear mechanism of action does not diminish the neuroprotective potential of TD139 in glaucoma.

## Electronic supplementary material

Below is the link to the electronic supplementary material.

Supplementary Material 1

## Data Availability

The datasets used and/or analysed during the current study are available from the corresponding author on reasonable request. All data generated or analysed during this study are included in this published article and its supplementary information files.

## Abbreviations

Gal 3: Galectin-3
GCL: Ganglion cell layer
GFAP: Glial fibrillary acidic protein
IB4: Isolectin GS-IB4
INL: Inner nuclear layer
IOP: Intraocular pressure
IPL: Inner plexiform layer
NFL: Nerve fiber layer
OHT: Ocular hypertension
RBPMS: RNA-binding protein with multiple splicing
RGC: Retinal ganglion cell
