# Supplementary material for: Intravitreal injection of the Galectin-3 inhibitor TD139 provides neuroprotection in a rat model of ocular hypertensive glaucoma
Source: Mol Brain. 2024 Nov 22;17:84. doi: 10.1186/s13041-024-01160-z (PMC11583433; doi:10.1186/s13041-024-01160-z)
Supplement: Supplementary file 1 — Supplementary Material 1 [file 13041_2024_1160_MOESM1_ESM.pdf]

Supplementary materials

Methods

Table S1. Primary and secondary antibodies

**Antibodies**	**Working concentration**	**Product ID**	**Company**
Anti-RBPMS	0.002 mg/ml	NBP2-20112	Novus Biologicals
IB4 (biotinylated)	0.005 mg/ml	I21414	Invitrogen
Anti-GFAP	1:500	NBP1-05198	Novus Biologicals
Alexa Fluor 568 goat anti-rabbit	0.004 mg/ml	A11011	Invitrogen
Streptavidin 488	0.004 mg/ml	S11223	Invitrogen
Alexa Fluor 647 goat anti-chicken	0.004 mg/mL	AB150171	Invitrogen
Nuclei (Hoechst)	0.005 mg/mL	H1399	Invitrogen

Table S2. P-value and 95% CI per comparison of conditions for all statistical analyses.

	***P*-value**	**95% CI**
**IOP**		
Mean		
NCTRL – SHAM-HBSS	0.971	[-10.902, 7.469]
NCTRL – OHT-HBSS	< 0.001 ***	[10.625, 24.669]
NCTRL – SHAM-TD139	1.000	[-7.144, 7.511]
NCTRL – OHT-TD139	< 0.001 ***	[13.653, 25.847]
Peak		
NCTRL – SHAM-HBSS	0.993	[-9.370, 12.126]
NCTRL – OHT-HBSS	< 0.001 ***	[18.801, 35.242]
NCTRL – SHAM-TD139	0.828	[-5.795, 11.361]
NCTRL – OHT-TD139	< 0.001 ***	[18.944, 33.218]
AUC		
NCTRL – SHAM-HBSS	0.981	[-133.682, 95.715]
NCTRL – OHT-HBSS	< 0.001 ***	[153.695, 329.062]
NCTRL – SHAM-TD139	0.997	[-82.348, 100.648]
NCTRL – OHT-TD139	< 0.001 ***	[170.519, 322.781]
**RBPMS+ cell soma counts**		
NCTRL – SHAM-HBSS	0.558	-
NCTRL – OHT-HBSS	0.003 **	-
NCTRL – SHAM-TD139	0.609	-
NCTRL – OHT-TD139	0.656	-
**ISOB4+ microglia counts**		
NFL-GCL		
NCTRL – SHAM-HBSS	1.000	[-3.630, 3.940]
NCTRL – OHT-HBSS	0.011 *	[0.714, 6.500]
NCTRL – SHAM-TD139	0.385	[-1.311, 5.064]
NCTRL – OHT-TD139	0.003 **	[1.154, 6.257]
IPL-INL		
NCTRL – SHAM-HBSS	0.995	[-2.653, 3.351]
NCTRL – OHT-HBSS	0.085	[-0.214, 4.375]

NCTRL – SHAM-TD139	0.998	[-2.296, 2.759]
NCTRL – OHT-TD139	0.012 *	[0.478, 4.525]
**ISOB4+ microglia surface area measurements**		
NFL-GCL		
NCTRL – SHAM-HBSS	0.002 **	-
NCTRL – OHT-HBSS	< 0.001 ***	-
NCTRL – SHAM-TD139	0.089	-
NCTRL – OHT-TD139	< 0.001 ***	-
IPL-INL		
NCTRL – SHAM-HBSS	0.932	-
NCTRL – OHT-HBSS	0.012 *	-
NCTRL – SHAM-TD139	0.020 *	-
NCTRL – OHT-TD139	0.001 **	-
**ISOB4+ monocyte counts**		
NFL-GCL		
NCTRL – SHAM-HBSS	0.860	-
NCTRL – OHT-HBSS	0.860	-
NCTRL – SHAM-TD139	0.860	-
NCTRL – OHT-TD139	0.860	-
**GFAP volume**		
NFL-GCL		
NCTRL – SHAM-HBSS	0.970	[-5.2578, 3.601]
NCTRL – OHT-HBSS	0.040 *	[0.146, 7.287]
NCTRL – SHAM-TD139	0.957	[-4.535, 2.982]
NCTRL – OHT-TD139	0.019 *	[0.487, 6.592]

Results

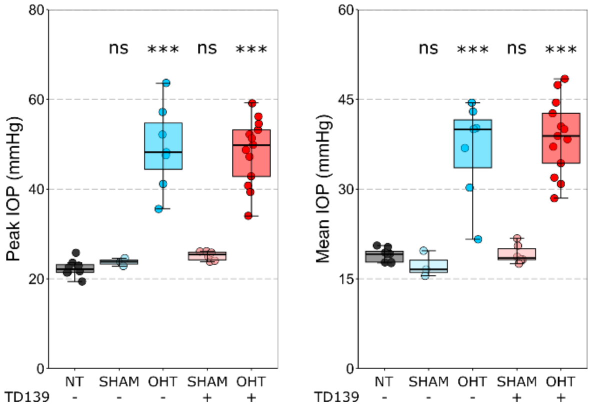

Figure S1. Peak (left) and mean (right) IOP of the rat groups. The OHT groups show a significantly higher peak and mean IOP compared to the control, showing that intravitreal injection did not disturb the induction of OHT. ns = $P > 0.05$, * = $P < 0.05$, ** = $P < 0.01$, *** = $P < 0.001$.
